# Words of Consolation

**Published:** 1886-10-16

**Authors:** 


### Words of Consolation.

Communications for this column should be addressed, "Chaplain," care of the Editor of The Hospital.

Patients who have sufficient faith to be sure, in their own minds, that God has a distinct purpose in laying them upon a sick-bed, have advanced further than they suppose in the way of consolation. It is a comfort, however much you suffer, to feel that you are in God's hands, and to know that He is doing His best for you. But it is a greater comfort if you can discern a good reason for your being called to suffer. We believe that a reason may be discerned by most sufferers who seek for it prayerfully. Their eyes will be gradually opened, and they will understand God better, perhaps, than they ever did before. At all events, we shall not be wrong in advising some who may read these lines to take a steady look back at their past lives, and then to ask themselves whether there is not mercy to be discerned in the very check now imposed upon them. Why has the ordinary life, with its labour and its busy running to and fro, come to a stop? If conscience here reminds you (as it may, possibly) of a past which has been absolutely sinful, or if you have been living without God in the world, there can be no doubt at all about God's purpose. It only remains to be seen whether you will fall in with it. How patient and long-suffering God has been with your soul! While you were in health He spoke to you and pleaded with you often, and you declined to listen. Well, then, is it to be wondered at that His love now urges Him to take stronger measures? Have you much difficulty in seeing that such measures are necessary, and that it might have been infinitely worse for you if they had not been ordained? God is always pleading with us; but we harden so terribly in the hour of our wealth, and yet so gradually and unconsciously, that nothing less than a rude shock brings us to our senses. Then the pleading of the Holy Spirit becomes perceptible again. The Divine voice, in the still hours of sickness, sounds plainer than ever. Oh, what hours those are for listening! In spite of pain and restlessness, and sleepless nights and tiresome days, there does come to most sufferers "the still, small voice," asking in some cases for repentance, in others for a more thorough consecration of life to God.

The main purpose you had in coming into hospital was to be cured of a physical disorder, or at least to obtain relief. We have seen that your Saviour may have a twofold purpose in bringing you here, even as He had in the days of old, when He healed the sick with a touch of His hand; and that maybe to say not only in His good time, "Arise and walk," but to lead you to desire the far greater blessing, "Thy sins be forgiven thee."

He prayeth well who loveth well
Both man and bird and beast;
He prayeth best who loveth best
All things both great and small;
For the dear God who loveth us,
He made and loveth all.—Coleridge.

